# Yuan-Zhi decoction in the treatment of Alzheimer’s disease: An integrated approach based on chemical profiling, network pharmacology, molecular docking and experimental evaluation

**DOI:** 10.3389/fphar.2022.893244

**Published:** 2022-08-24

**Authors:** Qiong Wu, Xiang Li, Xiao-Wen Jiang, Dong Yao, Li-Jun Zhou, Zi-Hua Xu, Nan Wang, Qing-Chun Zhao, Zhou Zhang

**Affiliations:** ^1^ Department of Life Science and Biochemistry, Shenyang Pharmaceutical University, Shenyang, China; ^2^ Department of Pharmacy, General Hospital of Northern Theater Command, Shenyang, China

**Keywords:** network pharmacology, molecular docking, molecular mechanism, Alzheimer’s disease, Yuan-Zhi decoction

## Abstract

Yuan-Zhi Decoction (YZD) is a traditional Chinese medical formulation with demonstrated clinical benefits in Alzheimer’s disease (AD). We used liquid chromatography coupled with mass spectrometry to identify 27 unique chemical components of YZD. Analyzing these using network pharmacology and molecular docking models identified 34 potential interacting molecular targets involved in 26 biochemical pathways. When tested in an animal model of AD, the APP/PS1 transgenic mice showed measurable improvements in spatial orientation and memory after the administration of YZD. These improvements coincided with significantly reduced deposition of Aβ plaques and tau protein in the hippocampi in the treated animals. In addition, a decreased BACE1 and beta-amyloid levels, a downregulation of the p-GSK-3β/GSK-3β, and an upregulation of the PI3K and p-AKT/AKT pathway was seen in YZD treated animals. These *in vivo* changes validated the involvement of molecular targets and pathways predicted in silico analysis of the chemical components of YZD. This study provides scientific support for the clinical use of YZD and justifies further investigations into its effects in AD. Furthermore, it demonstrates the utility of network pharmacology in elucidating the biochemical mechanisms underlying the beneficial effects of traditional Chinese medicines (TCM).

## Introduction

Alzheimer’s disease (AD) is an irreversible, chronic, and relentless neurodegenerative disease, characterized by memory loss, psychoses, affective disorders, and behavioral alterations (Gong et al., 2015; Fang et al., 2017; Cai F. F. et al., 2018; Pang et al., 2018; Sun et al., 2018). Numerous details of AD-related neuropathological changes have been described, including the formation of amyloid plaques, neurofibrillary tangles, and loss of neurons and synapses. Nonetheless, key events leading to the pathogenesis and progression of the disease remain poorly understood (Kumar et al., 2015).

Considering the multifactorial causation of AD, pharmacotherapy approaches targeting a single molecule may not optimal. And As an example, although the anti-amyloid antibody, aducanumab, developed by Biogen, has been shown to lower the number of amyloid plaques in the brain, there is no evidence that it improves cognitive outcomes. Thus, the accelerated approval of aducanumab by the FDA remains controversial (Mullard, 2021). Given the worldwide impact of AZ effective novel approaches to the management of the disease are urgently needed (Luo et al., 2020). TCM has been in clinical use for thousands of years, particularly in the management of chronic diseases. Numerous Chinese herbal formulations show efficacy in the treatment of AD. These are gaining gradual worldwide acceptance due their holistic approach acting simultaneously of multiple symptoms, while exhibiting limited side effects (Wang and Zhang, 2017).

Yuan-Zhi Decoction (YZD) is a natural plant-derived TCM formulation. It can be traced back to the book “Qi xiao liang fang” written by Fang Xian during the Ming Dynasty. YZD is a mixture of eight Chinese herbs, namely: *Polygala tenuifolia* Willd. (yuan-zhi in Chinese), *Panax ginseng* C. A. Mey. (ren-shen in Chinese), *Acorus tatarinowii* Schott (shi-chang-pu in Chinese), *Notopterygium incisum* Ting ex H. T. Chang (qiang-huo in Chinese), *Asrum heterotropoides* Fr. Schmidt var. *mandshuricum* (Maxim.) Kitag. (xi-xin in Chinese), *Ephedra sinica* Stapf (ma-huang in Chinese), *Paeonia lactiflora* Pall. (chi-shao in Chinese), *Atractylodes macrocephala* Koidz. (bai-zhu in Chinese). This formulation has been in use to treat a variety of medical issues. Recent pharmacological studies have demonstrated that many ingredients of YZD have beneficial effects in AD. Yuanzhi (*Polygala tenuifolia* Willd.) is an ingredient of multiple formulas use to managed age related memory impairment (May et al., 2016; Howes et al., 2017; Li B. et al., 2021). Shichangpu (Acori Tatarinowii Rhizoma), has pronounced neuro-pharmacological effects (Oh et al., 2004; Chang and Teng, 2018; Song et al., 2018; Wang L. et al., 2019; Deng et al., 2020). Xixin decoction was shown to reduce phosphorylation toxicity at specific sites of the tau protein in the brains of rats (Diwu et al., 2013). However, due to the complex composition of YZD, a comprehensive elucidation of the molecular mechanisms involved in tits beneficial effects in AD remains incomplete.

Network pharmacology, first proposed by Andrew Hopkins in 2007 is a relatively novel approach in elucidating the mechanism of action of multicomponent drugs (Luyen et al., 2015; Lee et al., 2018; Wang N. et al., 2019). Its core concept is to optimize treatment strategies based on the biological network of disease features, bioactive agents, and drug targets that are connected to each other (Hopkins, 2007). With rapid progress in bioinformatics and systems biology, network pharmacology is becoming an increasingly viable approach in identifying active compounds and elucidating the underlying pharmacological mechanisms involved in their beneficial effects (Li et al., 2011; Li and Zhang, 2013; Cai H. et al., 2018; Zhou et al., 2020). Through the integration of chemical component identification, target prediction, and network construction, this approach can effectively predict the molecular mechanisms affected by the active compounds in TCM formulations (Luo Z et al., 2020). As such network pharmacology represents a promising tool in the study of the mechanism of action of complex formulations containing a multitude of active ingredients (Li et al., 2020).

Molecular docking studies use computer simulations to study the interactions between receptors and small molecules potentially binding to them. By predicting certain features, such as the location and affinity of these interaction it is an invaluable tool in drug design and screening of chemical compounds (Chen et al., 2014; Lionta et al., 2014; Saikia and Bordoloi, 2019; Reddyrajula and Dalimba, 2020). Recently the combination of network pharmacology and molecular docking analysis was successfully applied to study the active component of TCM formulations (Wang Y. et al., 2019).

In the study presented here we systematically identified chemical constituents of YZD using liquid chromatography/mass spectrometry and explored possible targets and related pathways with potential relevance to the treatment of AD. The validity of this approach was confirmed by behavioral evaluation, microscopic analysis of the brains, and by Western blotting.

## Materials and methods

### Chemicals, reagents and materials

HPLC-grade acetonitrile was obtained from Merck KgaA (Darmstadt, Germany). HPLC-grade formic acid was supplied by Macklin Biochemical Co., Ltd. (Shanghai, China). Ultra-pure water (18 MΩ cm) was prepared *via* a Milli-Q water purification system (Milford, United States). 3,6′-disinapoyl sucrose, Ginsenoside Re, *β*-Asarone, Asarinin, Notopterol, Ephedrine, Paeoniflorin, and Atractylenolide II were purchased from National Institutes for Food and Drug Control (Beijing, China). The purity of each reference standard was above 98%. All eight herb constituents of YZD, including *Polygala tenuifolia* Willd., *Panax ginseng* C. A. Mey., *Acorus tatarinowii* Schott, *Notopterygium incisum* Ting ex H. T. Chang, *Asrum heterotropoides* Fr. Schmidt var. *mandshuricum* (Maxim.) Kitag., *Ephedra sinica* Stapf, *Paeonia lactiflora* Pall., and *Atractylodes macrocephala* Koidz. were purchased from the Hebei Renxin Pharmaceutical Co., LTD., and authenticated by Deputy Chief Pharmacist Jing He (Department of Pharmacy, General Hospital of Northern Theater Command). Their voucher specimens were deposited at the author’s laboratory.

### Preparation of YZD extract and standard solution

Raw herbs contained in the formula (yuan-zhi 9.3 g; ren-shen 18.6 g; shi-chang-pu 18.6 g; qiang-huo 18.6 g; xi-xin 18.6 g; ma-huang 18.6 g; chi-shao 37.3 g; bai-zhu 37.3 g) were soaked for 2 h and extracted by reflux extraction using a 10-fold mass of 70% ethanol aqueous solution. This process was repeated twice. Raw extracts were filtered with a 200-mesh sieve, combined, and concentrated under vacuum, resulting in 60 g thick paste. The extract was obtained with a yield of 33.7% and kept frozen at −20°C until use. A 1.0 g of this paste was sonicated in 50 ml of methanol/water mixture (1:1, v/v) for 5 min, centrifuged at 1,500×*g* for 10 min at 4°C, the supernatant was filtered through a 0.22 μm membrane filter, and used for further analysis.

### Chromatography/mass spectroscopy equipment and parameters

Chromatographic separation was carried out on a Thermo Ultimate 3000LC, Q Exactive HF LC-MS system (Thermo, United States) utilizing an Agilent Zorbax Eclipse C18 column (1.8 μm, 2.1 × 100 mm) at 30°C with a flow rate of 0.3 ml/min. The mobile phase was 0.1% formic acid in water (solvent A) and acetonitrile (solvent B). The gradient elution program was performed as follows: 0–2 min, 5% B; 2–6 min, 5–30% B; 6–7 min, 30% B; 7–12 min, 30–78% B; 12–14 min, 78% B; 14–17 min, 78–95% B; 17–20 min, 95% B; 20–21 min, 95–5% B; 21–25 min, 5% B. 2 μL of sample solution was injected for analysis. Mass detection was performed using an Thermo Ultimate 3000LC Q Exactive HF mass spectrometer (Thermo, United States) equipped with a ESI source operating in both positive and negative mode with the following operating parameters: drying gas (N2) temperature: 325°C, sheath gas flow rate: 45 arb; auxiliary gas flow rate: 15 arb, purge gas flow rate: 1 arb, electrospray voltage: 3.5 KV, capillary temperature: 330°C, S-Lens RF Level: 55% under positive or negative mode. The nozzle voltage was set at +500 V or −1000 V, respectively. The quasimolecular ions [M-H]^−^ and [M + H]^+^ were selected as precursor ions and subjected to target-MS/MS analyses with different collision energies ranging from 10 to 60 V to acquire sufficient product ions. MS spectra were recorded over the m/z range of 50–1,000. The Compound Discoverer 3.1 platform was used to perform retention time correction, peak identification, peak extraction, and other tasks. Data was processed using the Thermo mzCloud online database and Thermo mzVault local database to identify substances based on the secondary mass spectrum information.

### Network pharmacology analysis

#### Establishing the database of YZD components

Briefly, all available Chinese medicine network pharmacology research databases (TCMSP, TCMID, ETCM) were searched for previously identified chemical components of YZD. We also prepared a list a list of all chemical components identified by combined LC-MS analysis. This comprehensive list of YZD components was used to search the PubChem (https://www.ncbi.nlm.nih.gov/pubmed/), SEA (https://sea.bkslab.org), STITCH (https://stitch.embl.de/), PharmMapper (https://lilab-ecust.cn/pharmmapper/), and SwissTarget Prediction databases (http://www.swisstargetprediction.ch/) to create a comprehensive list of molecules present in YZD that could mediate beneficial effects in AD.

#### Establishing the AD therapeutic target database

To identify potential AD-related therapeutic targets we conducted multiple database searches with the search term “Alzheimer’s Disease” and limiting searches to “*Homo sapiens*”. The following databases were queried: Drugbank database (https://www.drugbank.ca/); GeneCards database (https://www.genecards.org/); OMIM database (http://omim.org/); DisGeNET database (http://www.disgenet.org/), and GEO database (https://www.ncbi.nlm.nih.gov/geo/). Potential AD-related molecular targets identified from these searches were merged into a single list and duplicates were removed, resulting in a list of potential drug targets in AD.

#### Protein-protein interaction network analysis

The list of potential drug targets in AD (described in 2.4.2) was intersected with a list of known or presumed molecular constituents of YZD (described in 2.4.1) using the Omic Share Tools. Molecules present in both lists represented molecules in YZD that were likely to be responsible for its beneficial effects in AD. The Omic Share Tools were used to draw Venn diagrams.

To clarify relationships between the proteins identified in this process their protein-protein interaction (PPI) network was constructed and analyzed using the STRING database (https://string-db.org/), with the species limited to *Homo sapiens*.

#### KEGG pathway enrichment analysis and Gene Ontology term performance

KEGG Pathway enrichment and Gene Ontology (GO) term performance analysis were conducted on proteins involved in the network using the Kyoto Encyclopedia of Genes and Genomes (KEGG, http://www.genome.jp/kegg/) (Ogata et al., 1999) database and the Database for Annotation, Visualization and Integrated Discovery (DAVID) system (http://david.abcc.ncifcrf.gov/home. jsp/, v6.7) (Dennis et al., 2003) Relevant pathways and GO terms with a *p* value < 0.05 were selected as significant in the development of AD.

#### Network construction and analysis

To facilitate the interpretation of the complex relationships between putative beneficial components of YZD and known or presumed therapeutic targets in AD, an interaction network was created using the Cytoscape software (version 3.6.0, Boston, MA, United States). This provided an approach for data integration, analysis, and visualization for complex network analysis. (Huang et al., 2019)

### Molecular docking analysis of potential active YZD ingredients

To further verify the reliability of the target prediction results, a group of key molecules was selected based on their importance in the“component-target-pathway” network. Three-dimensional structures of the selected target proteins were downloaded from the RCSB (http://www.rcsb.org) database and saved in pdb format. The Maestro software package was used to create a representation of these key proteins, including the removal of repeat units, hydrogenation and charge treatment. Then the docking site of various YZD-derived chemicals on the target proteins was determined, and a grid file was prepared. Models containing the selected protein and the interacting YZD chemical were crated by molecular docking, using the docking score (GlideScore, GS) as the scoring standard.

### Experimental evaluation

#### Animals and drug administration

Six-month-old male transgenic (APPswe PSEN1dE9) 85Dbo mice (APP/PS1) on a C57BL/6 background were obtained from Beijing HFK Bioscience Co. Ltd. These double transgenic animals express both a human-mouse chimeric APP gene (Mo/Hu APP695) carrying the Swedish mutation (Swedish KM594/595NL) and a human PS1 gene with a mutation in its ninth exon (dE9). APP/PS1 mice and wild-type littermates were genotyped by PCR analysis of genomic DNA from tail biopsies. Animals were maintained at 22 ± 2°C with a 12-h light-dark cycle (lights on at 8 a.m., lights off at 8 p.m.) with free access to food and water. All experiments were approved by the Animal Care and Use Institutional Committee of the General Hospital of the Northern Theater Command. APP/PS1 transgenic AD mice were randomly divided into five groups. Animals in the three YZD treatment groups received 0.11 g/kg, 0.32 g/kg, or 0.96 g/kg YZD. Two control groups were given either 4 mg/kg Donazepil or water. A group of wild type mice (WT), sham treated with the same volume of water was used as an additional control group. Treatment or sham treatment was administered *via* oral gavage for 3 months.

#### Morris water maze test

After the 3 months treatment period the Morris water maze (MWM) test was used to assess cognitive behavior. The test consisted of a one-day conditioning test, a five-day spatial acquisition test, and a one-day probe trial test. Tests were performed in a circular pool (120 cm in diameter and 50 cm in height) filled with water at 20–22°C. The pool was filled to a depth of 26 cm with water containing an opaque nontoxic Titanium dioxide die, and placed in a dimly lit, soundproof test room surrounded by distinct extra maze cues. The pool was equally divided into four quadrants. A white hidden circular platform (12 cm in diameter and 25 cm high) was placed randomly under a quadrant of the pool, submerged 1 cm below the surface of the water. In the conditioning test, mice swam freely in the pool for 60 s. Each mouse was placed in the pool in the four different quadrants every day. The timer was set to 60 s and automatically stopped once the mouse found the platform and remained on it for 5 s. Animals unable to locate the platform themselves withing 60 s were manually moved to the platform and allowed to stay on it for 20 s. The probe trial test was performed on the 7th day when the platform was removed. Mice were allowed to swim for 60 s, searching for the missing platform at its last know location. The escape latency, distance, speed, and the number of times animals crossed the platform were recorded using a video camera-based Supermaze System (Shanghai Xinruan Information Technology Co., Ltd., Shanghai, China).

#### Western blot analysis

Mice were anesthetized with sodium pentobarbital (50 mg/kg), killed, and brains were harvested for Western blot analysis. Hippocampi, isolated from the brain, were homogenized in RIPA lysis buffer (Solarbio Science & Technology Co., Beijing, China) containing a protease and phosphatase inhibitor cocktail (Thermo Scientific, Waltham, MA, United States). The homogenized tissue was centrifuged at 12,000 rpm for 10 min at 4°C to remove debris and the protein concentration of each sample was determined using a BCA protein assay (Solarbio Science & Technology Co.). The prepared homogenates were used to estimate the protein expression levels of APP, BACE1, beta-amyloid, pGSK3B/GSK3B, and pAKT/AKT. Samples were electrophoresed using SDS-10% PAGE, transferred to a PVDF membrane, and protein expression was determined using antibodies against APP, BACE1, beta-amyloid, pGSK3B/GSK3B, pAKT/AKT, and actin (1:1,000; Abcam, Boston, United States), respectively. Signal was detected using an ECL method and quantified in a ChemiDoc MP Imaging System (Bio-Rad, California, United States).

**FIGURE 1:**
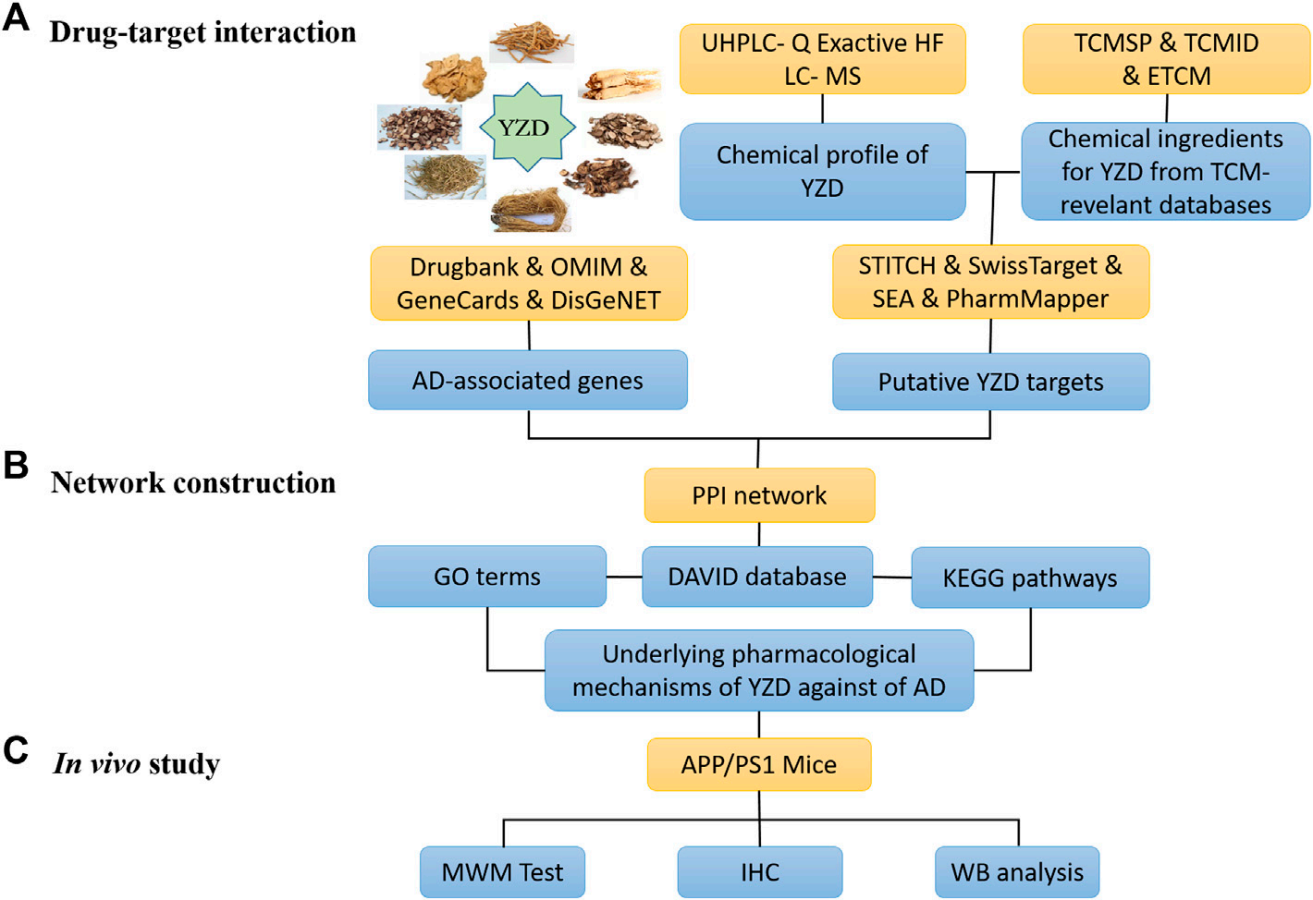
Flowchart of the steps taken during the system pharmacology analysis of the therapeutic mechanisms of action of Yuan-Zhi Decoction (YZD) in Alzheimer’s disease (AD).

The schematic diagram depicting the steps of all experimental work is shown in Figure 1.

### Immunohistochemical analyses

Hemispheres prepared for immunohistochemical analyses were immersion fixed in 5% paraformaldehyde, and paraffin embedded brain samples were cut into 3 μm slices using a microtome (Leica RM2235). Slides were deparaffinize with graded ethanol, incubated with the primary antibody overnight at 4°C and with the appropriate fluoroprobe labelled secondary antibody for 1 h at room temperature, protected from light. Antibodies used were: Anti-Amyloid beta 40 Rabbit (Servicebio, GB111197) (1:500) for the detection of Aβ plaques, and anti-Tau Rabbit (Servicebio, GB111178) (1:200) for Tau.

### Statistical analysis

The experimental data were analyzed using SPSS 20.0 package (SPSS Inc., Chicago, IL, United States). Figures were created by GraphPad Prism 6.0 software (GraphPad Software Inc., San Diego, CA, United States). All results are expressed as mean ± standard error of the mean (SEM). Multigroup comparisons in the MWM test were performed using two-way ANOVA. For other experimental data, one-way ANOVA was used, using LSD to test for homogeneity of variance. A non-parametric test was used for unequal variance. A *p* value <0.05 was considered statistically significant.

## Results

### Identifying chemical compounds present in YZD

**FIGURE 2:**
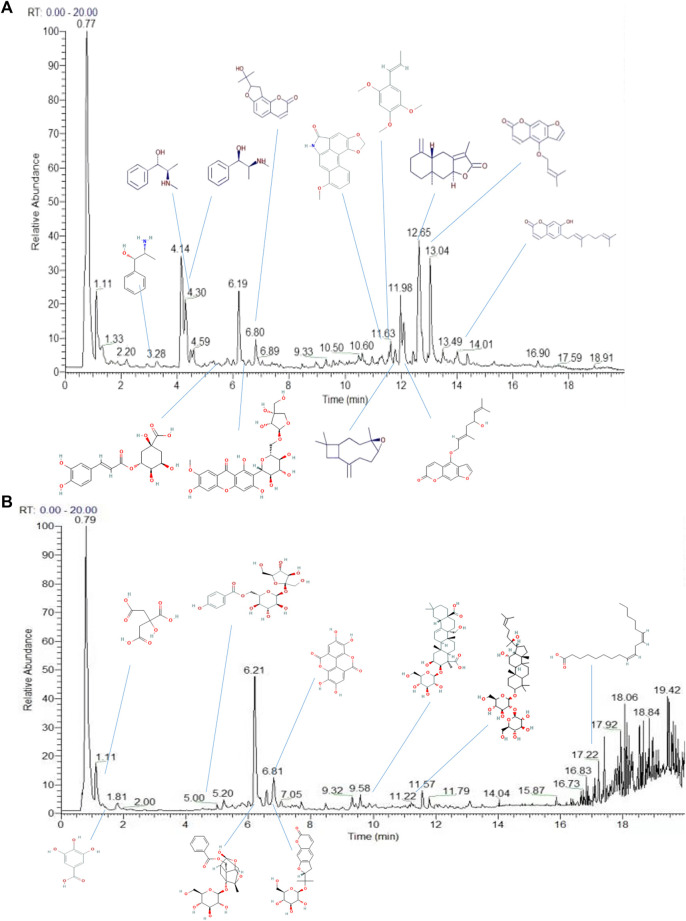
Total ion chromatograms of the chemical constituents of YZD in positive **(A)** and negative **(B)** ion modes during UHPLC-Q Exactive HF analysis.

Using a combination of liquid chromatography and mass spectroscopy identified 211 constituents of YZD based on mass, chromatographic behavior, MS/MS data, and fragmentation rules (Figure 2 and Supplementary Table S1 ). Additional data mining of databases relevant to TCM revealed a total of 1026 individual compounds present in YZD (Supplementary Table S2 ). Of these, 27 represented chemical components uniquely present in YZD, listed in Table 1. It is notable that of the 27 chemical ingredients, three compounds were found to be present in more than one herb.

**TABLE 1:** The main active ingredients of YZD.

Ingredients	CAS	Chemical formula
Polygalaxanthone	162857-78-5	C_25_H_28_O_15_
Tenuifolin	20183-47-5	C_36_H_56_O_12_
Ferulaldehyde	458-36-6	C_10_H_10_O_3_
Sibiricose A3	139726-39-9	C_19_H_26_O_13_
Citric acid	77-92-9	C_6_H_8_O_7_
Ginsenoside-Rg3	14197-60-5	C_42_H_72_O_13_
Ginsenoside-Rg_1_	22427-39-0	C_42_H_72_O_14_
Ginsenoside-Re	52286-59-6	C_48_H_82_O_18_
Ginsenoside-Rb_1_	41753-43-9	C_54_H_92_O_23_
Caryophyllene oxide	1139-30-6	C_15_H_24_O
Aristolactam I	13395-02-3	C_17_H_11_NO_4_
β-asarone	494-40-6	C_12_H_16_O_3_
Ephedrine	299-42-3	C_10_H_15_NO
Pseudoephedrine	90-82-4	C_10_H_15_NO
Norephedrine	37577-28-9	C_9_H_13_NO
linoleic acid	60-33-3	C_18_H_32_O_2_
Paeoniflorin	23180-57-6	C_23_H_28_O_11_
Gallic acid	149-91-7	C_7_H_6_O_5_
Ellagic acid	476-66-4	C_14_H_6_O_8_
Albiflorin	39011-90-0	C_23_H_28_O_11_
chlorogenic acid	327-97-9	C_16_H_18_O_9_
Notopterol	88206-46-6	C_21_H_22_O_5_
Isoimperatorin	482-45-1	C_16_H_14_O_4_
Columbianetin	3804-70-4	C_14_H_14_O_4_
Nodakenin	495-31-8	C_20_H_24_O_9_
Ostruthin	148-83-4	C_19_H_22_O_3_
Atractylenolide II	73069-14-4	C_15_H_20_O_2_

### Putative targets of the chemical constituents of YZD

Based on database searches we found that the 27 key components of YZD had the potential to interact with a total of 949 predicted cellular targets. The contribution of the individual herbs is as follows: Polygalaxanthone: 12 targets, Tenuifolin: 43 targets, Ferulaldehyde: 238 targets, Sibiricose A3: 152 targets, citric acid: 376 targets, Ginsenoside-Rg_3_: 370 targets, Ginsenoside-Rg_1_: 102 targets, Ginsenoside-Re: 103 targets, Ginsenoside-Rb_1_: 114 targets, Caryophyllene oxide: 285 targets, *β*-asarone: 339 targets, Aristolactam I: 113 targets, Ephedrine: 109 targets, Pseudoephedrine: 48 targets, Norephedrine: 88 targets, linoleic acid: 546 targets, Paeoniflorin: 77 targets, gallic acid: 434 targets, ellagic acid: 479 targets, albiflorin: 426 targets, chlorogenic acid: 424 targets, Notopterol: 362 targets, Isoimperatorin: 419 targets, Columbianetin: 265 targets, Nodakenin 327: targets, Ostruthin: 474 targets, and Atractylenolide II: 48 targets. After combining and deduplicating the list of potential interaction partners, resulted in 949 predicted targets. Detailed information of these is given in Supplementary Table S3.

### Known AD-related therapeutic targets

Database searches identified a total of 231 cellular molecular targets with presumed or proven relevance to AD. Of these 31 were found in the DrugBank database, 87 in the GeneCards database, 43 in the DisGeNET database, and 25 in the OMIM database. After removing duplicates resulted in 169 AD-related targets. The details of these, including additional information, are given in Supplementary Table S4. We also looked for genes specifically up- or downregulated in AD at mRNA level. The GSE1297 and GSE36980 chip series from the GEO database were selected for this analysis. These chipsets were submitted to the GEO2R online analysis tool that identified 65 co-expressed, differentially regulated genes that are potentially relevant to AD. These mRNAs were matched to universal gene names from the Uniprot database. The results of this analysis are shown in Supplementary Table S5.

### Network and pathway analysis

#### YZD chemical component—Disease target intersection

**FIGURE 3:**
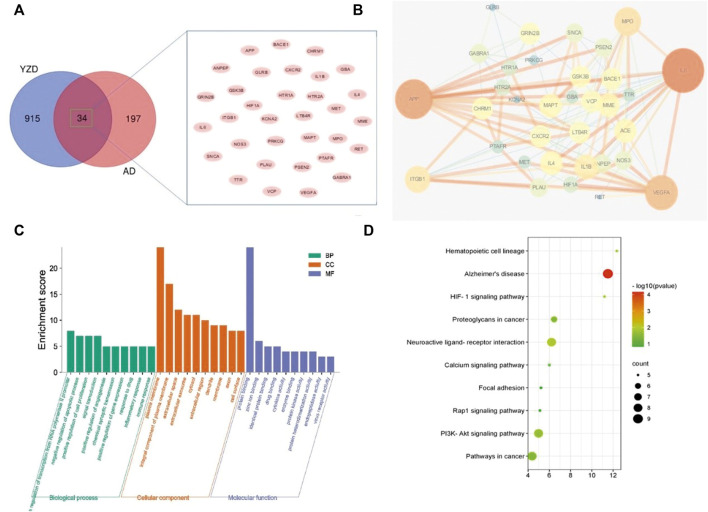
Analysis of proteins potentially affected by YZD treatment. **(A)** Venn diagram illustrating the overlap between proteins interacting with components of YZD and proteins know or predicted to be involved in the pathogenesis of AD. **(B)** PPI network of AD-related proteins that can interact with YZD components. **(C)** Analysis of the GO classification of the same set of proteins. **(D)** KEGG enrichment results of the interacting proteins represented as a bubble map. Red indicates greater, green a lesser enrichment of a given KEGG pathway, while the size of the bubbles corresponds to the number of proteins in each group.

We compared the 949 know molecular targets of the active ingredients of YZD with the 231 AD-related differentially expressed mRNAs identified in Section 3.3. As shown in the Venn diagram (Figure 3A) 34 molecules were present in the overlap area. These molecules, representing targets in AD on which the chemical constituents of YZD are likely to act, are listed in Table 2.

**TABLE 2:** The common target of drugs and diseases.

Name	Number	Common targets
The common target of drugs and diseases	34	ACE, ANPEP, APP, BACE1, CHRM1, CXCR2, GBA, GLRB, GRIN2B, GSK3B, HIF1A, HTR1A, HTR2A, IL1B, IL4, IL6, ITGB1, KCNA2, LTB4R, MAPT, MET, MME, MPO, NOS3, PLAU, PRKCG, PSEN2, PTAFR, RET, SNCA, TTR, VCP, VEGFA, GABRA1

#### Construction of protein-protein interaction network map and selection of key targets

To investigate protein-protein interaction (PPI) relationships between the identified 34 molecules, a PPI network was built using the String platform and Cytoscape. This network included 34 nodes and 137 edges, with an average degree value of 8.06, and a PPI enrichment *p* value of less than 1.0e^−16^. Importing the interaction relationship data into the Cytoscape 3.6.0 software resulted in the PPI network map shown in Figure 3B. The calculated average shortest path length, betweenness centrality, closeness centrality, and degree of nodes in the network are shown in Table 3. According to network topology properties, the targets are sorted from high to low (degree score≥8) with larger degree values indicating the greater relevance of a node within the network. Sorting molecules accordingly identified 17 key molecules: IL6, APP, VEGFA, MPO, ITGB1, IL1B, ACE, MME, MAPT, IL4, CXCR2, GRIN2B, VCP, GSK3B, LTB4R, CHRM1, and BACE1. These proteins may represent the critical targets of YZD in the prevention and treatment of AD.

**TABLE 3:** Topological parameters of the targets.

name	Degree	Closeness centrality	Betweenness centrality
IL6	22	0.717391	0.174759
APP	19	0.702128	0.211938
VEGFA	16	0.647059	0.111052
MPO	12	0.559322	0.021201
ITGB1	11	0.589286	0.064417
IL1B	10	0.559322	0.014098
ACE	9	0.55	0.008529
MME	9	0.55	0.019121
MAPT	9	0.568966	0.023683
IL4	9	0.540984	0.013697
CXCR2	8	0.559322	0.027057
GRIN2B	8	0.5	0.046652
VCP	8	0.540984	0.018205
GSK3B	8	0.568966	0.020185
LTB4R	8	0.559322	0.024173
CHRM1	8	0.532258	0.031334
BACE1	8	0.540984	0.012885

#### Functional analysis based on GO classification

To investigate further the biological process (BP), molecular function (MF), and cellular components (CC) of these key proteins, GO enrichment analysis was carried out. The top 10 GO terms (*p* < 0.05) were selected as significant entries based on their *p* value (Figure 3C). This GO category analysis indicated that the majority of hubs were involved in transcription of RNA polymerase II promoters, apoptosis, cell proliferation, and signal transduction. Classifying proteins based on molecular functions, they were involved in protein binding, zinc ion binding, and drug binding, while they were situated in various cellular locations, being associated with the plasma membrane, forming integral components of plasma membrane, or being present in the extracellular space.

#### KEGG pathway enrichment analysis

KEGG pathway enrichment analysis demonstrated that the 34 key targets contributed to 26 distinct pathways (*p* value <0.05). The top 10 of these are listed in Table 4 and Figure 3D. The most prominently represented KEGG pathways contained pathways associated with, Neuroactive ligand-receptor interaction, and the PI3K-Akt signaling pathway.

**TABLE 4:** The enriched KEGG pathway and the related genes.

Pathway	Genes	Fold enrichment	*p* Value
Alzheimer’s disease	BACE1, GSK3B, APP, MME, IL1B, PSEN2, MAPT, GRIN2B, SNCA	11.52	0.0000
Neuroactive ligand-receptor interaction	GABRA1, CHRM1, GLRB, PTAFR, HTR1A, HTR2A, GRIN2B, LTB4R	6.21	0.0002
PI3K-Akt signaling pathway	ITGB1, IL4, GSK3B, IL6, CHRM1, NOS3, MET, VEGFA	4.98	0.0007
Pathways in cancer	ITGB1, RET, PRKCG, GSK3B, IL6, HIF1A, MET, VEGFA	4.38	0.0015
Proteoglycans in cancer	ITGB1, PRKCG, PLAU, HIF1A, MET, VEGFA	6.45	0.0018
Hematopoietic cell lineage	IL4, IL6, MME, IL1B, ANPEP	12.35	0.0006
HIF-1 signaling pathway	PRKCG, IL6, NOS3, HIF1A, VEGFA	11.20	0.0008
Calcium signaling pathway	PRKCG, CHRM1, NOS3, PTAFR, HTR2A	6.00	0.0081
Focal adhesion	ITGB1, PRKCG, GSK3B, MET, VEGFA	5.22	0.0130
Rap1 signaling pathway	ITGB1, PRKCG, GRIN2B, MET, VEGFA	5.12	0.0139

#### Construction of a network illustrating the roles of herb components in molecular pathways and their interacting proteins

**FIGURE 4:**
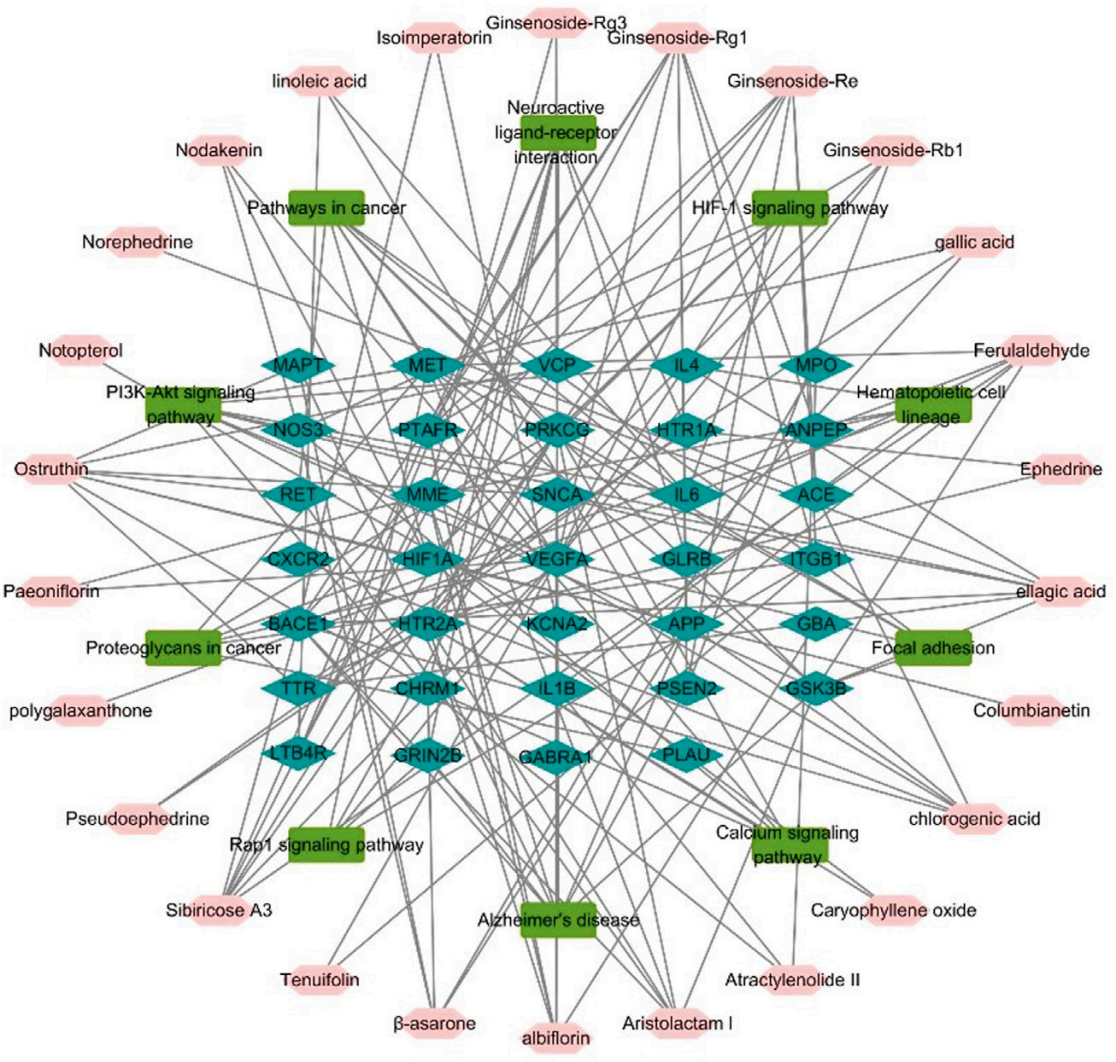
Herb component—putative molecular target - major pathway network map. Pink nodes represent active ingredients of YZD, blue nodes represent potential targets, and the green nodes indicate the involved KEGG pathways.

To represent the relationship between key chemicals components of YZD, their putative molecular targets, and the molecular pathways they are involved in a herb component - putative molecular target - major pathway network map was constructed using Cytoscape 3.6.0 (Figure 4). This map illustrates clearly the relationship between the 27 key constituents of YZD, the 10 most prominent pathways identified in KEGG enrichment analysis, and the 34 AD-related proteins identified in Section 3.4. This mapping illustrated that the main pathways in the action of YZD in AD belong to KEGG pathways associated with Alzheimer’s disease, PI3K-Akt signaling, and Neuroactive ligand-receptor interactions (Table 4).

### Molecular docking analysis

**FIGURE 5:**
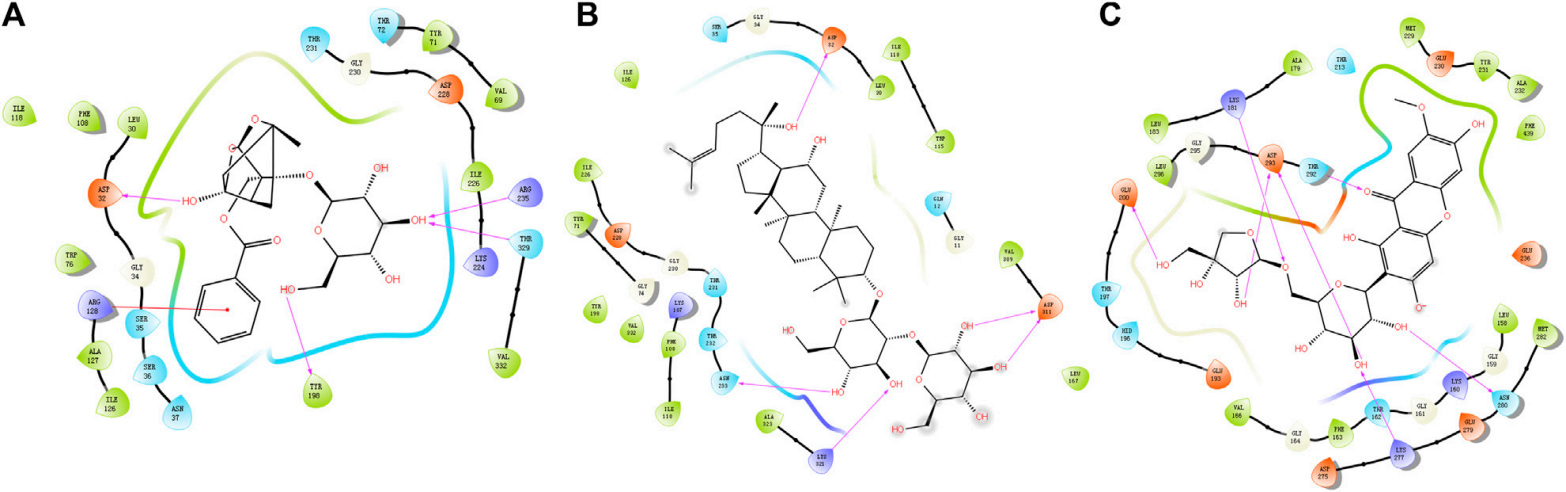
Molecular docking models depicting interactions between YZD components and their putative molecular targets/receptors. **(A)** APP (Beta amyloid A4 protein Beta, PDB code: 6UWP) interacting with Paeoniflorin; **(B)** BACE (Beta-secretase 1, PDB code: 5CLM) interacting with Ginsenoside Rg3; **(C)** GSK3B (Glycogen synthase kinase-3 beta, PDB code: 1O6L) with Polygalaxanthone. Structures drawn entirely in black represent YZD components, the colored dots are amino acid residues from the interacting protein. Pink arrows represent hydrogen bonds while red lines indicate a P-π conjugate.

Available crystal structures of identified key molecular targets were downloaded and the Maestro platform was utilized to analyze their docking relationship with chemical components identified from YZD. We selected 8 proteins from the herb component - putative molecular target - major pathway network map for molecular docking analysis. Their interactions with the 27 key compounds of YZD were used as docking partners. In the resulting models a lower the score indicates higher binding energy, with a more stable conformation in ligand-receptor binding. This in turn increases the possibility of interaction in vitro and *in vivo* biological systems. The results of molecular docking (Figure 5; Table 5) and Glide energy analysis (Table 6) demonstrated strong binding between the active components of YZD and the 8 proteins selected from the herb component - putative molecular target - major pathway network map. These results provide further verification of the predicted interactions between YZD components and molecular targets. Figure 5 shows data obtained from three docking analysis models. Interactions between APP and Paeoniflorin occur through hydrogen bonds *via* amino acid residues ASP32, TYR198, ARG235, THR329, and a p-π conjugate is formed with Arg 128 (Figure 5A). BACE1 forms stable hydrogen bonds with Ginsenoside Rg3 *via* amino acid residues ASP32, ASN233, Lys321, ASP311 (Figure 5B), while there are stable hydrogen bonds between GSK3β and Polygalaxanthone *via* amino acid residues Glu200, ASP293, Lys181, and ASN 280 Figure 5C).

**TABLE 5:** The docking information of 8 targets with 27 compounds of YZD.

	IL6	APP	VEGFA	MPO	IL1B	ACE	GSK3B	BACE1
Citric acid	−5.9	−3.1	−2.6	−6.6	−4.4	−3.9	−4.2	−4.7
Gallic acid	−6.5	−5.3	−4.1	−5.5	−5.4	−6.3	−5.1	−5.1
Pseudoephedrine	−5.3	−6.6	−4.7	−4.1	−5.1	−6.6	−5.4	−5.6
Ephedrine	−5.4	−6.4	−4.3	−4.0	−5.3	−7.9	−5.7	−5.6
Ferulaldehyde	—	−4.5	−3.2	—	−5.0	—	−5.8	−4.9
Norephedrine	−6.2	−6.7	−5.1	−4.7	−5.6	−8.0	−6.5	−6.4
Isoimperatorin	−3.7	−4.5	−3.0	−3.6	−4.6	−6.7	−5.1	−4.4
Nodakenin	−5.5	−5.4	−3.7	−3.2	−4.2	−7.2	−5.6	−5.9
Columbianetin					−5.2	−8.4	−6.2	−4.4
Aristolactam I	−4.0	−4.4	−2.7	−4.3	−4.6	−7.5	−4.4	−4.8
Ginsenoside-Re	—	—	—	—	−3.5	—	—	−5.9
Ginsenoside-Rg1	—	—	—	—	−5.9	—	−5.6	−4.7
Paeoniflorin	−2.3	−4.7	−2.1	−2.7	−5.1	−6.4	−5.0	−5.1
Caryophyllene oxide		−4.6		−3.7	−4.4	−6.0	−4.3	−4.2
Chlorogenic acid	−5.5	−5.4	−4.6	−5.4	−4.9	−5.9	−7.4	−4.3
Linoleic acid	−3.0	−0.7	0.9	−1.5	0.7	−2.9	−0.5	−1.1
Ostruthin	−4.5	−5.6	−2.9	−3.2	−4.1	−6.9	−5.6	−6.0
β-asarone	−3.9	−4.4	−3.1	−3.5	−4.5	−5.4	−4.6	−3.2
albiflorin	−4.1	−5.3	−2.7	−4.8	−5.2	−8.5	−5.2	−5.4
Notopterol	−3.7	−5.1	—	−3.9	−5.3	−6.4	−5.4	−5.1
Ginsenoside-Rg3	−4.1	—	—	—	−4.1	−8.4		−5.0
Polygalaxanthone		−6.5	−1.0	−4.2	−5.1	−7.9	−7.3	−4.6
Atractylenolide II	−3.1	−5.3	—	−4.1	−4.6	−6.6	−6.1	−4.0
Ellagic acid	−2.6	−3.6	−0.9	−2.5	−3.5	−6.4	−3.8	−2.8
Ginsenoside-Rb1	—	—	—	—	−3.6	—		−4.8
Tenuifoli	—	—	—	—	−3.6	—	−3.1	−4.4
Sibiricose A3	−2.5	−6.8	−4.3	−4.4	−6.5	−8.7	−7.8	−5.5

**TABLE 6:** The Glide energy information of 8 targets with 27 compounds of YZD.

	IL6	APP	VEGFA	MPO	IL1B	ACE	GSK3B	BACE1
Citric acid	−22.0	−12.7	−9.3	−29.7	−22.2	−22.3	−23.4	−21.0
Gallic acid	−21.4	−22.5	−17.4	−25.3	−23.8	−18.1	−27.0	−23.0
Pseudoephedrine	−20.8	−30.0	−18.8	−20.9	−24.2	−29.7	−27.3	−25.9
Ephedrine	−21.5	−28.4	−18.5	−19.1	−22.7	−30.7	−27.8	−27.4
Ferulaldehyde	−25.3	−26.9	−18.0	−25.0	−26.2	−32.3	−33.0	−28.3
Norephedrine	−22.3	−25.5	−20.7	−21.7	−24.6	−28.4	−24.9	−24.3
Isoimperatorin	−22.4	−29.8	−21.1	−23.8	−28.7	−36.3	−35.4	−30.0
Nodakenin	−37.7	−46.4	−27.5	−31.6	−38.9	−46.2	−52.4	−48.8
Columbianetin	—	—	—	—	−27.2	−31.0	−37.6	−31.6
Aristolactam I	−22.3	−32.5	−16.9	−25.2	−28.7	−35.6	−34.1	−32.1
Ginsenoside-Re	—	—	—	—	−36.2	—	—	−56.1
Ginsenoside-Rg1	—	—	—	—	−51.9	−43.2	−57.2	−49.2
Paeoniflorin	−24.4	−44.7	−26.5	−35.7	−40.8	−35.5	−54.4	−46.2
Caryophyllene oxide	—	−25.9	—	−22.4	−23.3	−27.5	−27.3	−25.0
Chlorogenic acid	−33.0	−42.7	−34.3	−43.1	−39.3	−40.0	−48.8	−40.2
Linoleic acid	−26.7	−33.2	−21.4	−27.3	−25.1	−38.6	−33.3	−35.7
Ostruthin	−26.5	−37.2	−22.3	−28.3	−32.1	−42.1	−41.4	−38.9
β-asarone	−18.9	−26.4	−14.9	−22.0	−22.8	−27.2	−27.3	−22.9
Albiflorin	−23.1	−36.5	−15.1	−26.9	−31.6	−24.5	−39.0	−31.9
Notopterol	−28.5	−39.9	—	−31.5	−36.9	−42.2	−42.4	−40.1
Ginsenoside-Rg3	−36.4	—	—	—	−39.4	−48.0	—	−51.5
Polygalaxanthone	—	−66.9	−25.8	−44.9	−3.9	−53.2	−74.2	−55.3
Atractylenolide II	−12.8	−26.2		−21.9	−21.6	−27.2	−27.9	−22.1
Elagic acid	−24.6	−40.1	−17.3	−29.7	−34.2	−40.9	−42.2	−38.1
Ginsenoside-Rb1	—	—	—	—	−38.3	—	—	−50.2
Tenuifoli	—	—	—	—	−34.0	−35.2	−24.7	−44.1
Sibiricose A3	−29.2	−58.9	−34.2	−40.5	−52.0	−52.7	−60.3	−50.7

### Studies in biological systems

#### YZD administration has beneficial effects on learning and memory in APP/PS1 mice

**FIGURE 6:**
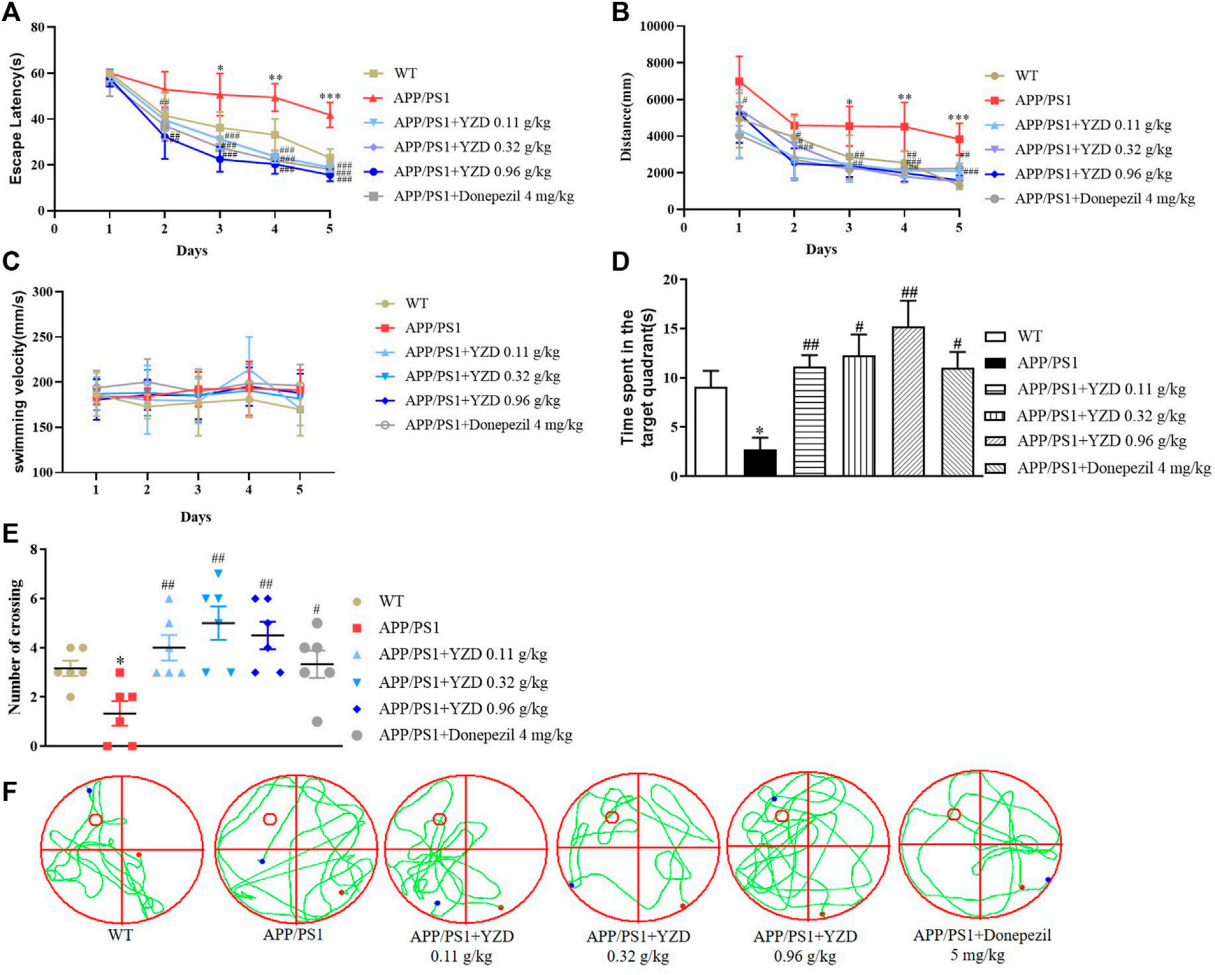
YZD administration ameliorated cognitive impairment in APP/PS1 mice. WT: wild-type mice; APP/PS1: APP/PS1 mice. **(A)** Swimming speed of mice in the Morris water maze. **(B)** Changes of escape latency of mice from the first day to the fifth day in different groups. **(C)** Changes in swimming distance from the first day to the fifth day in different groups. **(D)** The time that the indicated groups of mice spent at the target area that previously contained an escape platform in the Morris water maze. **(E)** The times of indicated groups of mice crossing the target area that previously contained an escape platform in the Morris water maze experiment. **(F)** Typical swimming trajectory maps of mice passing through the platform during the test period. *p < 0.05 compared to the WT group, *#p* < 0.05, #*#p* < 0.01 compared to the APP/PS1 group. All data are presented as mean ± SE (*n* = 6).

The effects of YZD on spatial learning and memory deficits in APP/PS1 transgenic mouse model were investigated in MWM trials. While there was no difference in swimming velocity (Figure 6A), APP/PS1 mice showed a significantly longer latency finding the hidden platform compared to their WT littermates. However, the administration of YZD at different doses to the transgenic animals significantly shortened the latency period (Figure 6B). Swimming distance was also reduced in the YZD treated groups (Figure 6C). Probe trials were performed to assess memory retention after the last training session. Compared to the WT group APP/PS1 mice showed a smaller number of intersections in the target quadrant and spent less time at the target area previously containing the escape platform (Figures 6D,E). YZD-treated APP/PS1 mice performed better, spending more time at the target area (Figure 6D). The number of crossovers in the YZD treated APP/PS1 mice was also greater than that of untreated controls (Figure 6E). Figure 6F shows typical swimming trajectory maps of mice passing over the platform during the test period. Taken together, these data suggest that YZD administration provided beneficial effects on learning and memory in APP/PS1 mice.

#### Western blot analysis

**FIGURE 7:**
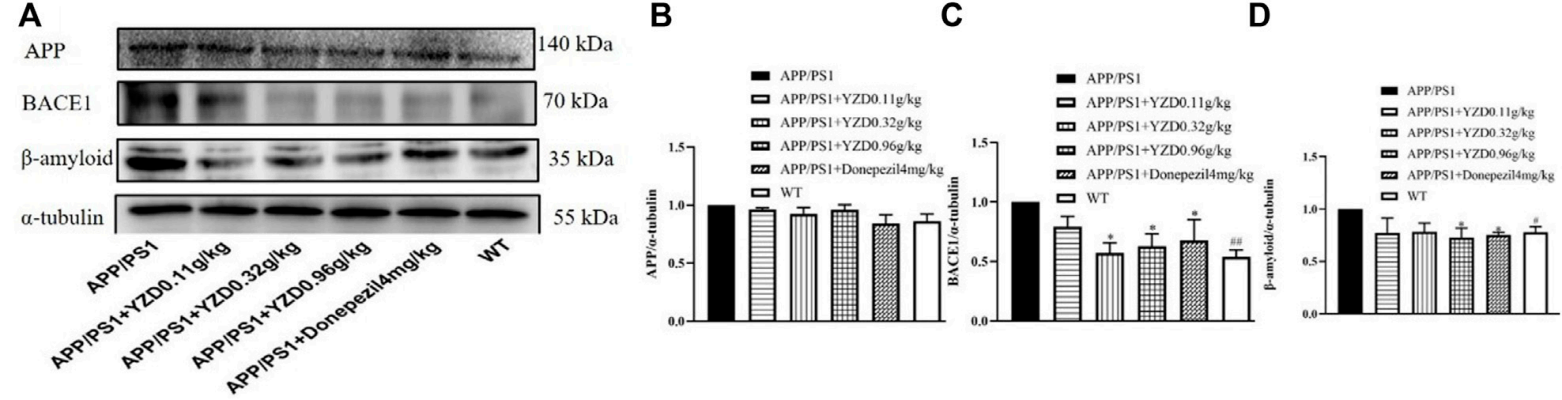
YZD treatment for 3 months reduces BACE1 and beta-amyloid protein abundance in the hippocampi of APP/PS1 transgenic mice. **(A)** Western blot analysis of APP, BACE1, beta-amyloid levels. Densitometry data of APP **(B)**, BACE1 **(C)** and beta-amyloid **(D)**. Values represented as the mean ± SE (*n* = 3), *p < 0.05, **p < 0.01, #*p* < 0.05 was considered statistically significant. Distinct symbols indicate differences in the level of significance between groups.

The hippocampi of animals from the MWM test were analyzed for the abundance of several AD-related proteins using Western blot analysis. As shown in Figure 7, while the abundance of APP appear different this change does not reach statistical significance (Figure 7A). However, the abundance of BACE1 (Figure 7B) and beta-amyloid (Figure 7C) expression were markedly down-regulated in APP/PS1 mice (*p* < 0.05). As expected, YZD and Donazepil (DON) treatment significantly decreased BACE1 expression (Figure 7B) and beta-amyloid (Figure 7C) levels compared to the untreated APP/PS1 group (*p* < 0.05). These results suggest that the therapeutic effect of YZD in AD may be attributed to the inhibition of BACE1 expression and the reduced production of beta-amyloid.

**FIGURE 8:**
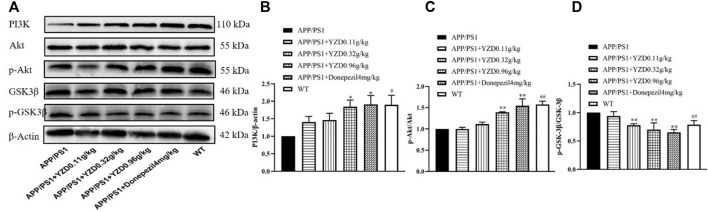
Effects of 3-months of YZD treatment on the expression of PI3K/AKT/GSK-3β pathway related proteins in the hippocampi of APP/PS1 mice. **(A)** Western blot analysis of PI3K, AKT, p-AKT, GSK-3β, p-GSK-3β. **(B)** Densitometry of PI3K, **(C)** Densitometry of p-AKT/AKT, **(D)** Densitometry of p-GSK-3β/GSK-3β. Values show the mean ± SE (*n* = 3), differences in the level of significance are: *p < 0.05, **p < 0.01, #*#p* < 0.01.

The PI3K/AKT/GSK-3β pathway correlates closely with the progression of AD. Therefore, we explored whether YZD had any effect on the expression of these proteins. Figure 8 shows a representative Western blot of PI3K, AKT, pAKT, GSK-3β and pGSK-3β levels. When compared to the WT group, the levels of PI3K (Figure 8A and p-AKT/AKT (Figure 8B) was significantly reduced in the hippocampi of untreated transgenic animals, while the levels of p-GSK-3β/GSK-3β increased (Figure 8C), with all observed differences reaching statistical significance (at least *p* < 0.05). Compared to untreated transgenic animals, the levels of PI3K (Figure 8A) and p-AKT/AKT PI3K (Figure 8B) increased in the groups receiving high doses YZD. Simultaneously, the levels of p-GSK-3β/GSK-3βPI3K (Figure 8C) decreased in the medium and high dose YZD treatment groups. The above results suggest that the therapeutic effect of YZD on AD may be related to the PI3K/AKT/GSK-3β signaling.

#### YZD treatment inhibited βAmyloid deposition and NFT in APP/PS1 transgenic mice

**FIGURE 9:**
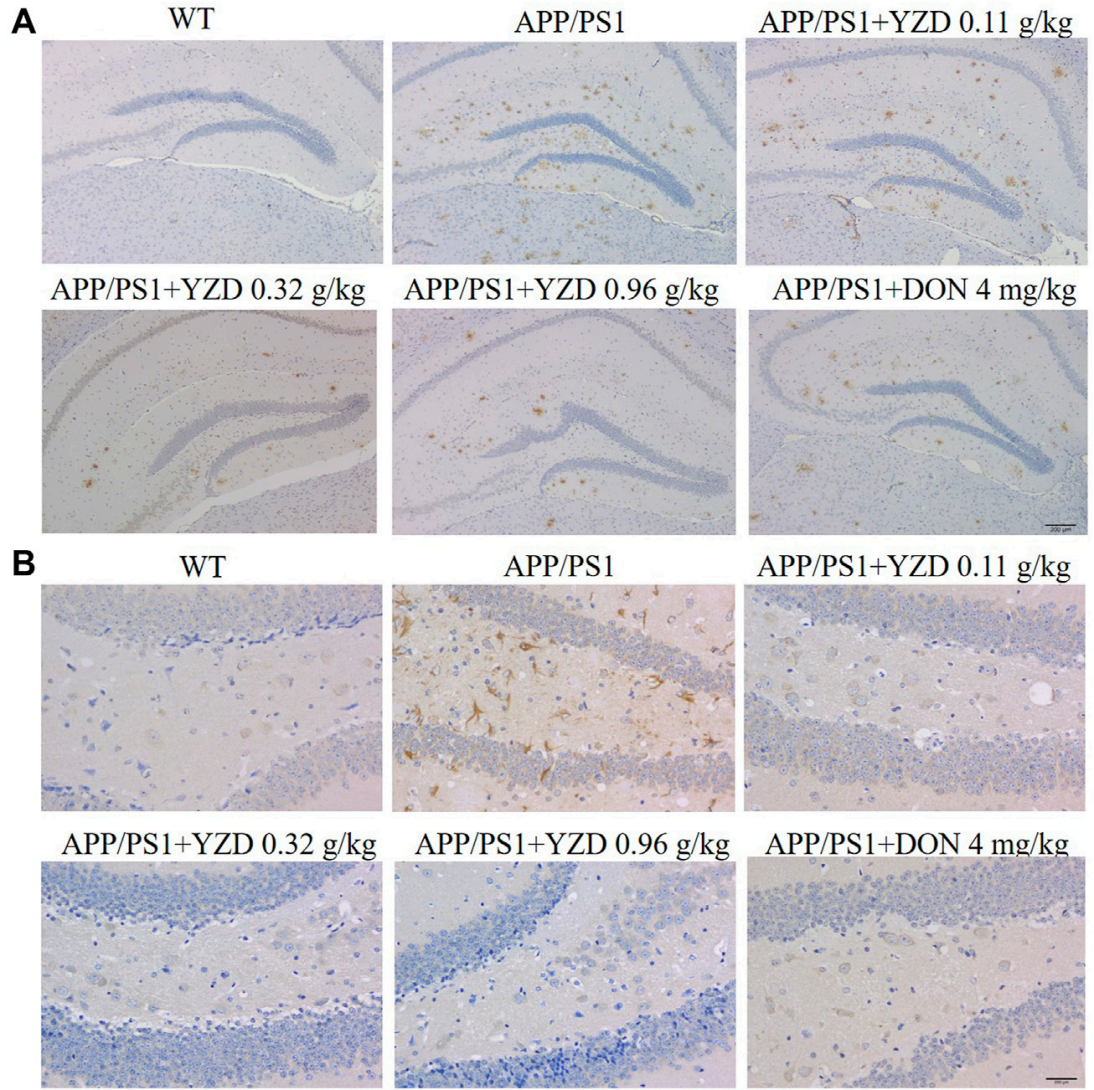
YZD inhibited Aβ and Tau deposition in the hippocampi of APP/PS1 mice. **(A)** Representative images of Aβ deposits (showing brown staining) in the hippocampi. Magnification: ×25, scale bar: 200 μm. **(B)** Images of Tau deposits in the hippocampi (appearing as brown deposits). Magnification: 400 × scale bar: 200 μm.

β Amyloid (Aβ) accumulation is a pathological feature of AD, inducing neuronal amyloidosis and even apoptosis. Therefore, we studied the effect of YZD on Aβ accumulation in the brain of APP/PS1 mice by immunohistochemistry. The resulting images shown numerous brown plaques. Tau protein, the main protein constituent of microtubule structure, maintains the stability of the microtubular system. In the pathogenesis of AD, microtubules depolymerize, resulting in the production of NFTs and the acceleration of the aging of neuronal fibers. Our results showed that the deposition of Aβ plaques was significantly decreased in the hippocampi of YZD-treated APP/PS1 transgenic mice (Figure 9). A similar reduction was seen in the deposition of Tau in the studied hippocampi.

## Discussion

AD is a complex disease affecting multiple biochemical pathways. Given its unclear etiology and pathogenesis, there is an increasing interest in the use of traditional medical formulations in the treatment of the condition (Poornima et al., 2016). TCM generally uses natural products, with a good safety record and low toxicity demonstrated during extensive periods of clinical use. TCM also relies on multi-compound formulations affecting several targets and multi-component pathways. Ultimately, this approach may have significant advantages over single compound medications in the treatment of complex, multifactorial diseases (Poornima et al., 2016).

To the best of our knowledge, this is the first study to systematically investigate the mechanism of action of YZD in combatting AD using network pharmacology analysis. The main chemical constituents of YZD were identified using liquid chromatography coupled to mass spectroscopy. This was followed by network pharmacology studies to assess potential interactions between the detected chemical components and putative AD-related protein targets identified in database searches. With this approach, both the protein targets and candidate active constituents of YZD were predicted in silico. By comparing the list of all potential target proteins affected by the constituents of YZD with a comprehensive list of proteins relevant to the development of AD we identified those proteins YZD is likely to act to deliver its beneficial effects in AD. Next, pathway enrichment analysis was performed to investigate the molecular pathways affected by the 27 components of YZD that were predicted act in AD. Following this computational phase of the work, the APP1/PS1 transgenic mouse model of AD was used to assess the *in vivo* efficacy of YZD in improving learning and memory deficits. These experiments demonstrated that YZD administration had a positive impact on spatial learning and memory, evidenced by reducing the time needed to reach a hidden platform during MWM trials. Finally, some of the network pharmacology predictions were validated by Western blot analysis and immunohistochemistry, confirming that several AD-related pathways predicted in silico indeed played a critical role in a live animal model.

In this study, data from UHPLC-Q Exactive HF mass spectrometry was complemented with TCM database searches to maximize the number of identified chemical constituents of YZD (Chu et al., 2016). In total 211 compounds were identified experimentally, including high levels of Prenol lipids, Phenol ethers, Benzopyrans, Benzene and substituted derivatives. Searches conducted in TCM-relevant databases found 1026 chemical compounds (Supplementary Table S2). Out of these two lists, a total of 27 compounds were identified that were uniquely present in YZD. Four of these were derived from the herb YuanZhi, 5 from RenShen, 2 from ShiChangPu, 6 from QiangHuo, 4 from MaHuang, 3 from XiXin, 4 from ChiShaoYao, and a single compound from BaiZhu, with 3 ingredients being present in more than one herb.

Oligosaccharide esters are a unique class of sugar esters in Polygonum consisting of a sucrose core and connect monosaccharides, mainly glucose and rhamnose, linked with different glycosidic bonds (Zhao et al., 2020). This raises the possibility that oligosaccharide esters may be biologically active substances in Polygonum japonica that could be utilized in the development of new anti-aging and neuroprotective drugs. While oligosaccharide esters exist in a variety of plants, a sugar core containing three or more monosaccharides can only be found in Polygala. It has been suggested that these unique oligosaccharide esters may have unusual pharmacologic effects (Li, 2008). Studies have shown that tenuifoliside A, tenuifoliside C (E), tenuifoliose B (D), tenuifoliside K, and tenuifoliside I (J) are reasonably well absorbed and enter the systemic circulation, exerting biological effects (Wang et al., 2017).

Some of the identified YZD components were previously shown to be beneficial in AD. Tenuifolin attenuates Amyloid-β_42_-induced neuroinflammation (Wei, 2016; Wang L. et al., 2019; Chen and Jia, 2020). This mechanism was protective against neuronal apoptosis and improved learning and memory deficits in a mouse AD model (Wang N. et al., 2019). Ginsenoside Rg1 significantly improved cognitive behavioral impairments in several animal models of AD (Nie et al., 2017; Liang et al., 2021), while ginsenoside Rg2 had a protective effect against memory impairments and neuronal death seen in Aβ25-35 transgenic rats (Cui J. et al., 2017; Cui et al., 2021). Ginsenoside Rg3 prevented cognitive impairment in a rat AD model (Zhang et al., 2019) and Notopterol effectively ameliorated cognitive deficits in APP/PS1 mice by attenuating pathology caused by the accumulation of amyloid-β and tau (Jiang X. et al., 2020; Jiang X. W. et al., 2020). Other studies have shown that Paeoniflorin exerted neuroprotective effects in a transgenic mouse model of AD (Zhang et al., 2015; Gu et al., 2016; Ma et al., 2018; Huang et al., 2020; Kong et al., 2020). Albiflorin ameliorated memory deficits in APP/PS1 transgenic mice (Xu et al., 2019) and *Atractylodes macrocephala* Koidz. was also used to treat Alzheimer’s disease (Zhu et al., 2018). *β*-Asarone, a crucial compound of YZD, was reported to prevent amyloid *β*-induced apoptosis by regulating the PI3K/AKT pathway in PC-12 cells (Li Z. et al., 2021; Meng et al., 2021). *β*-Asarone could also inhibit Amyloid deposition and reverse cognitive deficits in experimental animals (Wang et al., 2021). Nodakenin exhibited potent inhibitory activities against the *β*-site amyloid precursor protein cleaving enzyme 1 (BACE1) and is regarded as a promising therapeutic option in the treatment of AD (Yousof Ali et al., 2015).

Network pharmacology analysis is able to identify interactions between chemical constituents and their targets/pathways, and establish “compound-target-pathway” maps identifying chemical constituents and the mechanisms associated with their pharmacological activity (Ren et al., 2018). Our work is the first to explore the possible actions of YZD in AD using network pharmacology, laying the foundation for the use of this approach in further research. The potential pharmacological targets of the 27 YZD-derived compounds were identified with the help of the SEA, STITCH, Swiss Target Prediction database searches and PharmMapper. At the same time, protein molecules associated with the pathogenesis of AD were identified from the Drugbank, OMIM, GeneCards, and DisGeNET databases and by the analysis of differentially expressed genes in GEO datasets. This work identified 34 target molecules in AD that could be affected by ingredients in YZD.

After network topology analysis 17 of these genes, including IL6, APP, VEGFA, MPO, ITGB1, IL1B, ACE, MME, MAPT, IL4, CXCR2, GRIN2B, VCP, GSK3B, LTB4R, CHRM1, and BACE1 were considered to be the key molecules targeted by the chemical constituents of YZD. These proteins show complex interactions with each other, forming a PPI network. In such networks a change affecting a single molecule can result in a series of functional abnormalities, potentially leading to the emergence of pathologic abnormalities (Gu et al., 2020). Our results showed that APP, IL6, VEGFA, GSK3B and BACE1 could extensively interact with other members of the network. For example, APP interacts with BACE1, GSK3B, IL6, and PSEN2. APP is a widely expressed transmembrane protein playing a key role in the formation of Aβ in the pathogenesis of AD (Zhu et al., 2017). BACE1 is the main rate-limiting enzyme in the production of Aβ. Previous studies experimentally validated BACE1 as a YZD target in the treatment of AD (Ahmad et al., 2017; Prati et al., 2017). Furthermore, there is emerging evidence that BACE1 inhibitors may be potentially interesting tools in the management of AD (Rueeger et al., 2021), while the inhibition of GSK3B can reduce the phosphorylation of Tau and the conversion of APP to Aβ. These observations provide strong evidence that several components of YZD have significant association with various AD-related signaling pathways. Our molecular docking models investigating the interactions between validated targets (i.e., APP, BACE1 and GSK3B) and the 27 identified key chemical compounds found in YZD provides additional support for a role of YZD in the treatment of AD.

KEGG pathway enrichment analysis showed that the identified molecules preferentially affected the transcription of RNA polymerase II promoters, the process of apoptosis, cell proliferation, signal transduction, synaptic transmission, neuroactive ligand-receptor interactions, and the PI3K-Akt, HIF-1, and calcium signaling pathways. The PI3K/AKT signaling pathway is involved in regulating cell proliferation, differentiation, metabolism, and apoptosis. It also exerts anti-oxidative, anti-inflammatory, and neuroprotective effects. Altered regulation of this pathway has been found to play a central role in the development of AD (Cui W. et al., 2017; Razani et al., 2021). PI3K activation can act as a second messenger to transmit signals into cells, activating AKT phosphorylation. In turn, AKT could exert its anti-apoptotic effects by promoting the phosphorylation of downstream substrates, such as GSK-3β and mTOR. However, it was found that the activation of GSK-3β could lead to an abnormal increase in the level of phosphorylation of tau protein (Jimenez et al., 2011). Gene ontology analysis has also demonstrated that the molecular targets of YZD show a varied distribution within cells.

We also conducted behavioral tests to assess the efficacy of YZD in treating learning and memory deficits in aged APP/PS1 transgenic mice. These mice exhibit impairments in spatial learning and memory, as evidenced by their poor performance in MWM tests. Our results showed that YZD treatment restored learning and memory deficits in these animals, demonstrating a protective effect of this formulation in APP/PS1 transgenic AD mice.

The PI3K/AKT/GSK-3β signaling pathway plays a crucial role in cell growth, survival, apoptosis, and proliferation, and is also one of the important pathways that lead to the phosphorylation of tau protein and then the development of AD. (Kitagishi et al., 2014) Activation of PI3K will promote the phosphorylation of Thr308 within AKT, activating the protein. Thus, the activity of PI3K is reflected by the phosphorylation status of AKT. Activated AKT inhibits the action of GSK-3β downstream in this signaling pathway. GSK-3β is normally in the activated state in the cytoplasm of neurons. The activation of AKT results in the phosphorylation of the Ser9 residue of GSK-3β, inhibiting the activity of GSK-3β. GSK-3β is an important threonine/serine protein kinase, which can promote the phosphorylation of tau protein at multiple sites. (Kitagishi et al., 2014).

Our Western blot experiments indicated that treatment with YZD promoted the expression of PI3K and p-Akt, and reduced the levels of p-GSK3β, proving that YZD can regulate the activated PI3K/AKT/GSK3β pathway. The expression of BACE1 and *β*-amyloid were also down-regulated significantly, providing experimental confirmation of predictions reached by using network pharmacology and molecular docking analyses. Based on these findings, we propose that YZD may protect against the progression of AD by inhibiting the expression of BACE1, thereby reducing the production of beta-amyloid. The other main action of the formulation is *via* the regulation of the PI3K/AKT/GSK-3β pathway.

Although this work shed light on some of the therapeutic mechanisms of YZD in AD, the results need to be interpreted with caution. Additional pharmacologic experiments will be necessary to validate further mechanisms of YZD predicted to occur in our network analysis. Second, network pharmacology fails to identify dose-effect relationships of multi-component formulations. These issues will be addressed in subsequent studies. Finally, further work is needed to study global metabolic changes in other species to fully elucidate the mechanisms by which YZD improves AD. Enhanced metabolomics technologies, such as targeted metabolomics, are required to detect metabolite biomarkers (Wei et al., 2020).

**FIGURE 10:**
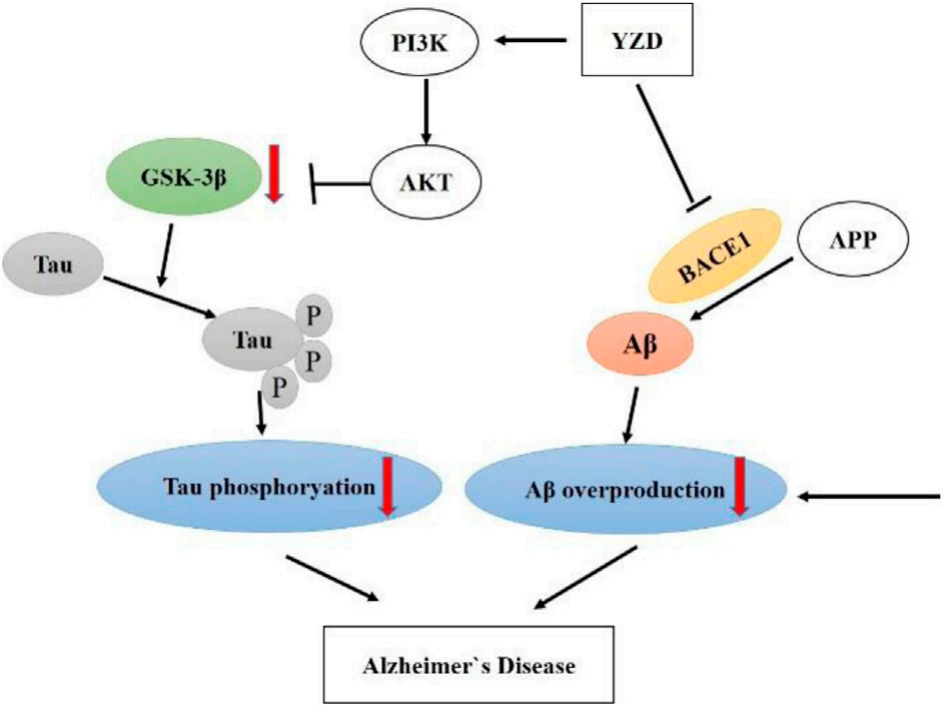
A schematic of proposed neuroprotective mechanism of YZD in AD.

In summary, multiple compounds in YZD show promise in the potential treatment of AD (Figure 10). Our experiments showed that YZD could reduce the expression of BACE1 and the production of beta-amyloid and regulate the PI3K/AKT/GSK-3β pathway. These findings are consistent with the results of our network pharmacology and molecular docking-based predictions. Thus, we propose that YZD is likely to affect a multitude of molecular mechanisms in the treatment of AD.

## Conclusion

Herbal medicines have provided valuable tools in preventing and treating AD for centuries. In this study, we have identified 27 chemical constituents of YZD. Using a network pharmacology approach combined with molecular docking analyses identified some of the mechanisms involved in the beneficial effects of YZD treatment in AD. These involve effects on neuroactive ligand-receptor interaction pathways, the PI3K/AKT signaling pathway, as well as HIF-1 and calcium signaling. The results showed that YZD has potential benefits on learning and memory deficits in APP/PS1 transgenic AD mice, and regulated the apoptotic process and signal transduction. The key proteins of related pathways were quantitatively analyzed using Western blots indicating that the extract of YZD could inhibit or activate key protein pathways. The insights provided by this preliminary work will pave the way for further studies into the potential mechanisms by which YZD acts as a therapeutic agent in AD.

## Data Availability

The original contributions presented in the study are included in the article/Supplementary Material, further inquiries can be directed to the corresponding authors.
